# Oomycete Communities Associated with Reed Die-Back Syndrome

**DOI:** 10.3389/fpls.2017.01550

**Published:** 2017-09-07

**Authors:** Martina Cerri, Rumakanta Sapkota, Andrea Coppi, Valentina Ferri, Bruno Foggi, Daniela Gigante, Lorenzo Lastrucci, Roberta Selvaggi, Roberto Venanzoni, Mogens Nicolaisen, Francesco Ferranti, Lara Reale

**Affiliations:** ^1^Department of Agricultural, Food and Environmental Sciences, University of Perugia Perugia, Italy; ^2^Department of Agroecology, Aarhus University Aarhus, Denmark; ^3^Department of Biology, University of Florence Florence, Italy; ^4^Department of Chemistry, Biology and Biotechnology, University of Perugia Perugia, Italy

**Keywords:** common reed, freshwater ecosystem, metabarcoding, microbial ecology, *Phragmites australis*, rhizosphere, wetlands

## Abstract

*Phragmites australis* (Cav.) Trin. ex Steud. die-back is a widely-studied phenomenon that was first discovered in northern Europe and that, until recently, was almost unknown in the Mediterranean basin. It has been described as a complex syndrome affecting reed populations leading to their retreat and decline, with significant impacts on valuable ecosystem services. Among the factors that cause the decline, soil-living microorganisms can be crucial. The aims of this study were to analyze the diversity of oomycetes communities associated with reed stands, and to understand whether they could play a key role in the decline. Variations in the structure of oomycetes communities were studied by metabarcoding of the internal transcribed spacer (ITS) 1 region of ribosomal DNA, from the sediments of five Italian freshwater ecosystems. They were chosen to cover a large variability in terms of surface area, water depth, microclimate, and presence of documented reed retreat. From 96 samples collected from reed roots, rhizosphere, and bulk soil, we assembled 207661 ITS1 reads into 523 OTUs. We demonstrated that oomycete communities were structured by several factors, among which the most important was die-back occurrence. Our study also indicates that *Pythiogeton* spp. could be potentially involved in the development of die-back. The role of heavy metals in the soil was also explored, and cadmium concentration was shown to affect oomycetes distribution. This study represents a significant step forward for the characterization of microbial communities associated with reed die-back syndrome and helps to gain knowledge of the complexity of these important wet ecosystems.

## Introduction

*Phragmites australis* is one of the most widespread angiosperms, with a cosmopolitan distribution range. It forms extensive stands in many types of aquatic habitats, especially in lakes and river shores, marshes, coastal brackish swamps, and lagoons (Engloner, 2009). The importance of this plant is widely acknowledged: it is used in phytoremediation (Guo et al., 2014), it protects the shoreline from bank erosion, and serves as a food resource or protection for arthropods, birds and mammals (Ostendorp et al., 2003). Even if this species sometimes shows an expansive behavior (Bertness et al., 2002; Foggi et al., 2011), since the 1950s an irreversible retreat of the population known as reed die-back syndrome (RDBS) has been observed from several wetlands of northern and central Europe (for a review, see van der Putten, 1997). In Italy, it was first detected in a brackish lagoon (Fogli et al., 2002), and recently in freshwater lakes (Gigante et al., 2011, 2014; Lastrucci et al., 2016a). Typical symptoms of RDBS include reduced plant height, weaker culms, abnormal rhizomes, formation of clumps (Armstrong et al., 1996; Fogli et al., 2002), and eventually retreat of the population (van der Putten, 1997). Some of the possible abiotic and biotic causes of reed decline has been widely investigated (Brix, 1999; Fogli et al., 2002). Among the abiotic factors, prolonged submersion of the plant appears to have dramatic consequences; Lastrucci et al. (2016a) demonstrated the existence of a relationship between reed die-back and permanent-artificially induced flooding conditions. Several macromorphological traits associated with RDBS (such as clumping habit, reduced culm height or diameter, high rate of dead buds, see Gigante et al., 2011, 2014; Lastrucci et al., 2016a for details) were significantly more often observed in permanently flooded stands than in the emerged stands. Furthermore, even if *P. australis* can tolerate high concentrations of heavy metals (Bonanno and Lo Giudice, 2010), some studies have highlighted their negative effect on plant fitness, suggesting a role in the RDBS (Gigante et al., 2014; Lastrucci et al., 2016a). Cd and Co, for example, seemed to be related to some traits associated with the die-back symptoms in Montepulciano (Lastrucci et al., 2016a).

The understanding of the possible biotic causes of RDBS is yet limited; the role of microorganisms living in the soil is still an issue of concern and deserves to be investigated, since plants are metaorganisms establishing close symbiotic relationships with their associated microorganisms (Mendes et al., 2013; Coats and Rumpho, 2014; Vandenkoornhuyse et al., 2015; Berg et al., 2016). To date, only a few studies have been carried out about the interaction between *P. australis* and bacteria or fungi (Wirsel et al., 2002; Neubert et al., 2006; Clay et al., 2016; Soares et al., 2016), and even less studies have explored the relationship between *P. australis* and communities of oomycetes in natural ecosystems (Nechwatal et al., 2008). Several oomycete pathogens have been shown to be involved in root disease complexes in many Poaceae (Toda et al., 2015; Grijalba et al., 2017). A previous study conducted in Lake Constance (Germany) suggested an association of the oomycete *Pythium phragmitis* with reed decline (Nechwatal et al., 2005). Therefore, we decided to investigate the involvement of oomycetes in RDBS in our study area.

Phylogenetic studies have shown that oomycetes are a diverse group of fungus-like eukaryotic microorganisms closely related to diatoms and sea weeds, and probably linked to the marine environment during evolution (Thines, 2014). They have colonized almost all ecosystems, from semi-arid regions (Mirzaee et al., 2013) to Arctic and Antarctic (Bridge et al., 2008) and include both saprophytes and pathogens of plants, insects, crustaceans, fish, vertebrate animals, and various microorganisms.

Culture-based techniques have allowed isolated microbes to be studied in detail, but they fail to represent the full microbial diversity inhabiting the natural environment, limiting the analysis to those that grow under laboratory conditions (Rondon et al., 2000; Fierer et al., 2007; Mendes et al., 2013). Molecular techniques, such as metabarcoding, overcomes these difficulties and allows the identification of microbes *in situ* (Ravin et al., 2015). Metabarcoding studies of oomycetes are limited in number and in efficacy, but recently, an improved strategy developed by Sapkota and Nicolaisen (2015) and based on a new annealing temperature, was demonstrated to successfully uncover the complexity of these microorganisms in soils.

The present study represents the first investigation of the diversity of reed-associated oomycetes; additionally, it is the first contribute to assess whether declining reed-beds harbor unique communities of oomycetes that differ from those of the healthy stands, and to understand the factors that influence their distribution. We evaluated the effect of the location, the die-back occurrence and submersion as driving forces in determining community composition. Henderson et al. (2017) suggested that heavy metals can inhibit the growth of oomycetes growth, being particularly toxic at zoospores stage, and with toxicity in order of Ag > Cu > Ni > Co > Zn; therefore, the effect of heavy metals on reed-associated oomycetes communities was investigated.

## Materials and Methods

### Site Description

**FIGURE 1:**
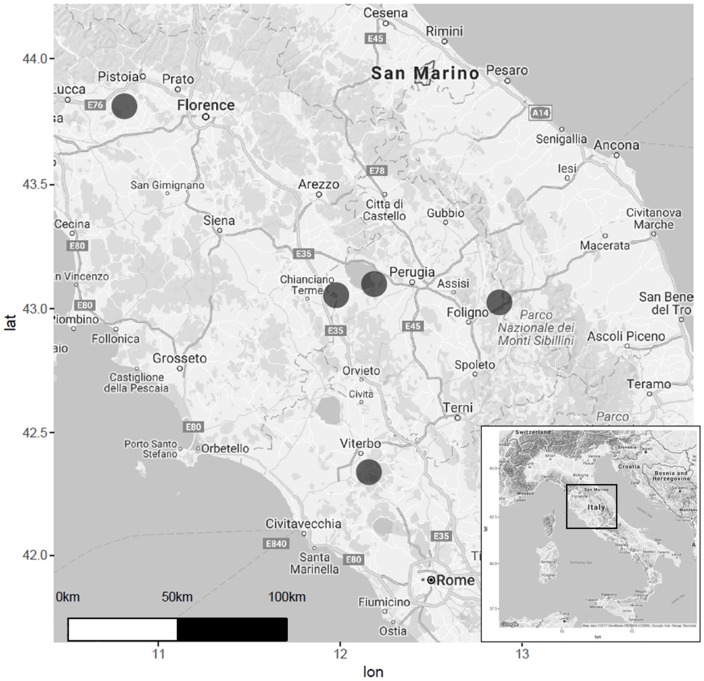
Map showing the distribution of Italian lakes selected for sampling.

Five wetlands from Central Italy were considered in this study: (1) Colfiorito Marsh, in Umbria Region (43°01′23″ N, 12°52′36″ E) at 756 m a.s.l.; (2) Lake Trasimeno, in Umbria Region (43°08′05.5″ N, 12°06′04.6″ E) at an average altitude of 257 m a.s.l.; (3) Lake Vico in Lazio Region (42°18′58.40″ N, 12°10′5.89″ E), 507 m a.s.l.; (4) Lake Chiusi in Tuscany Region (43°03′22.11″ N, 11°57′55.79″ E) at 252 m a.s.l.; (5) the wetland of “Le Morette” (43°48′30.38″ N, 10°48′20.14″ E) at Padule of Fucecchio Marsh, in Tuscany, at 13 m a.s.l. (**Figure 1**). These five ecosystems were chosen to cover a large variability in terms of: (i) surface area, from large lakes such as Trasimeno and Vico, to the small wetland of Le Morette and Colfiorito marshes; (ii) water depth, from the deep waters of Vico, to the shallow waters of Le Morette or Colfiorito marshes; (iii) microclimate, from temperate Apennine areas such as Colfiorito, to the submediterranean climate of Vico or Chiusi lakes; (iv) previous reed retreat documented by aerial images (Gigante and Venanzoni, 2012; Gigante et al., 2014).

### Soil and Roots Sampling

The sampling design was based on the results of former investigations in the same areas (Lastrucci et al., 2016b). Two types of stands could be identified for sampling: healthy stands (group N on Supplementary Table S1) and RDBS stands (group Y on Supplementary Table S1), the latter showing a number of RDBS diagnostic traits and, above all, the clumping habit. This is a clustered growth of the reed stems deriving from an uncontrolled outgrowth of dormant buds caused by the breaking of apical dominance, and represents a widely acknowledged and easily detectable trait of reed die-back (Armstrong et al., 1996; van der Putten, 1997; Clevering, 1998; Dinka and Szeglet, 2001; Gigante et al., 2011). Sampling was carried out during two different periods: in February 2015, bulk and rhizosphere soils were sampled from three healthy stands and three RDBS stands from four wetlands (Colfiorito, Trasimeno, Vico, and Chiusi) (48 samples); in September 2015, two healthy stands and two RDBS stands were sampled from all five wetlands, collecting, where possible, bulk, rhizosphere, and roots (48 samples). Bulk soil (40 g for molecular analysis and 300 g for chemical analysis) was collected at an approximate depth of 30 cm, 50 cm away from the plant, using a soil core sampler. Belowground part of the stem with roots were collected; roots were cut and thoroughly washed in distilled water for several times, while the soil attached to the roots was considered as rhizosphere. Soil and root samples were stored at -20°C until DNA extraction. For a summary of the samples used in this study, see Supplementary Table S1.

### DNA Extraction, DNA Amplification, and Pyrosequencing

Two different procedures were used for DNA extraction, one for soil samples (bulk soil and rhizosphere) and the second one for root samples. Soil samples weighing 40 g were mixed, freeze dried for 48 h, and ground; from these samples, 250 mg were used for total soil DNA extraction using the PowerLyzer^TM^ PowerSoil^®^ DNA Isolation Kit (Mo Bio Laboratories, Carlsbad, CA, United States) according to the manufacturer’s instructions, except that samples were homogenized in a Geno/Grinder 2000 (SPEX CertiPrep, Metuchen, NJ, United States) at 1500 rpm for three times, 30 s each. For root samples, 100 mg of fresh material were ground in a mortar with liquid nitrogen; DNA was extracted using the DNeasy Plant Mini kit (Quiagen GmbH, Hilden, Germany) according to the manufacturer’s instructions.

The internal transcribed spacer (ITS) 1 region of ribosomal DNA was used as a marker. Each sample was barcoded with 10-nucleotide multiplex identifier (MID) primer tags before pooling PCR products (Supplementary Table S1). Amplification strategy was as described by Sapkota and Nicolaisen (2015): the PCR reaction mixture consisted of 1× PCR reaction buffer, 1.5 mM of MgCl_2_, 0.2 mM of dNTPs, 1 μM of each primer, and 1 U of GoTaq Flexi polymerase (Promega Corporation, Madison, WI, United States) in a total volume of 25 μl containing 24 μl of reaction mixture and 1 μl of template. Tagged PCR amplicons from the 96 samples were pooled in equimolar amounts, electrophoresed on a 1.5% agarose gel and the visible smear of 200–400 base pairs was excised and purified from the gel using QIAquick Gel Extraction Kit (Quiagen, Hilden, Germany). The pooled samples were sequenced by Eurofins MWG (Ebersberg, Germany) on a GS Junior 454 Sequencer (Roche Diagnostics) using titanium chemistry. The raw data were deposited on the Sequence Read Archive website^1^ under the SRA number SRP111776.

### Sequence Analysis and Statistics

Sequences were analyzed using the bioinformatics pipeline available in QIIME v. 1.8 (Caporaso et al., 2010). De-multiplexing and quality filtering was carried out using split_libraries.py command using quality score of 50, discarding reads less than 150, homopolymer length of 10 nucleotides, maximum number of ambiguous bases of 6 and other default settings. The ITS1 region was extracted using ITSx extractor version 1.0.6 (Bengtsson-Palme et al., 2013). ITS1 reads were then clustered using the pick _otus.py script at 98% similarity level (Vettraino et al., 2012) using usearch61 with a minimum cluster size of two, thus excluding singletons (Edgar, 2010). For taxonomic assignment, a reference database described earlier (Sapkota and Nicolaisen, 2015) was used. In addition, for species identification, a representative sequence from each of the most abundant operational taxonomic unit (OTUs) with at least 100 reads in total was further subjected to Basic Local Alignment Search Tool (BLAST) matches at NCBI. At least the top five BLAST hits were evaluated: OTUs with identity and coverage ≥ 95% were assigned at species level, or at genus or family level if lower. Only reads assigned to oomycetes were retained in the OTU table.

The OTU table was used for diversity based calculations. Data visualization and statistical analysis were carried out in R (R Core Team, 2015). A species accumulation curve was generated using the *specaccum* function available in the ‘vegan’ package (Oksanen et al., 2015). Samples representing less than 400 reads were removed before performing diversity based calculations. Species richness and evenness were calculated by rarifying the OTU table at an even sampling depth of 400 reads, while the OTU table was transformed to relative abundance before beta diversity based calculations. The species evenness was evaluated by Pielou’s index and the species richness was estimated as the number of OTUs. β-diversity distance matrices were obtained using Bray–Curtis dissimilarity and visualized using principal coordinates analysis (PCoA) plot. Permutational multivariate analysis of variance (Anderson, 2001) with a permutation number of 999 was carried out to test the null hypothesis of no differences among *a priori* defined groups using the *adonis* function in ‘vegan’ package.

Covariance biplots were calculated using composition data analysis on centered log-ratio transformed OTU table (Gloor and Reid, 2016), removing samples less than 400 reads.

The OTU table was also used to perform the Dufrene-Legendre indicator species analysis in R, using the *labdsv* package (Roberts, 2012) to test the OTUs associated with die-back. OTUs with significant values (*P* < 0.05) and an indicator value > 0.25 were set as cut-off values to define indicator species (Dufrene and Legendre, 1997). The difference in relative abundance of OTUs was determined by non-parametric statistical method using Kruskal–Wallis test for ANOVA and Wilcoxon rank sum tests for pairwise comparison. The network analysis was made in QIIME using OTU table (with the command make_otu_network.py) and the resulting network files were visualized in cytoscape^2^. Samples and OTUs were subjected to network analysis via the spring-embedded algorithm using eweights, where samples represent larger nodes than OTUs. All OTUs are connected to the sample to which they belong with a line (edge). Shared OTUs are thus connected to several samples, and occupy the center of network, whereas OTUs that only occur in one or few samples occupy the periphery of the network. Samples are thus clustered based on shared OTU’s and the degree to which samples cluster is based on the number of OTUs shared between samples.

### Phylogenetic Analysis and Assessment of Taxa Associated to the RDBS

Using the indicator species analysis, 12 OTUs were found to have significantly higher abundance in die-back samples in the total dataset; they were aligned together with the ITS1 sequences of the accessions of *Pythiogeton* available in GenBank. ITS1 sequences of the two most abundant *Pythium* species in our dataset were used as outgroup. The Neighbor-joining tree was assembled with MEGA 7.0 (Kumar et al., 2015) and tested with a bootstrap of 1000 replicates, to ascertain the reliability of any given branch pattern.

### Determination of Heavy Metals in Soil and Plant Tissues

Concentrations of heavy metals (Cd, Pb, Zn, Cr, Ni, Cu, Al) in soil samples and in plant tissues were determined by Inductively Coupled Plasma Optical Emission Spectrometry (ICP-OES, Ultima 2, HORIBA Scientific) equipped with an ultrasonic nebulizer (U-5000AT, CETAC Technologies). The nebulizer gas flowrate was kept at 1 L min^-1^ and the plasma operated at a radiofrequency power of 1000 W. Supplementary Table S2 shows the wavelength used. Instrumental detection limits were in the range of 0.1–1.5 mg kg^-1^. Commercially produced (ICP multi-element standard solution IV CertiPUR^®^, VWR Merck Chemicals and Reagents) standard solutions (1000 mg L^-1^) in nitric acid were used to prepare appropriate elemental calibration standards. An acid digestion was performed on the soil (Ministero dell’Ambiente e della Tutela del Territorio [MATT] and Agenzia per la Protezione dell’Ambiente e per i Servizi Tecnici [APAT], 2005) and on the plant tissue samples (Du Laing et al., 2003) prior the analytical determination. The soils were air-dried, disaggregated using a mortar and pestle to pass through a 2 mm mesh sieve, dried at 105°C for 24 h and digested as follows: 8 ml of ultrapure nitric acid (Millipore Suprapur^®^, 65%) and 2 ml of ultrapure solution of hydrogen peroxide (Millipore Suprapur^®^, 30%) were added to 0.200 g of soil samples and digested in a Mars Microwave Oven, working at a power of 1000 W. Microwave digestion consisted of two steps: 130°C (200 psi) for 1 min, 180°C (300 psi) for 10 min. The mixture was cooled, filtered (Whatman Grade No. 42, particle retention 2.5 mm) and diluted with ultrapure water to 50 ml. Plant samples were preliminarily dissected into roots and rhizomes and dried at 70°C for 48 h. Acid digestion of tissues (0.4 g) was carried by the same procedure used for soils, but ultrapure water was added to reach the volume of 25 ml. The measure of the heavy metal was based on two replicates of the overall analytical procedure for each sample of soil and plant tissue examined.

## Results

### Data Structure and Taxonomic Composition

In total, 235065 reads were obtained from 454-pyrosequencing. After quality control, ITS extraction and taxonomic assignments, a total of 207661 reads were clustered into 523 OTUs assigned to oomycetes, excluding singletons. Two samples failed to yield any reads; thus, reads were obtained from 94 samples in total. The number of reads per sample revealed high variation (min, mean, max: 4, 2464, 13461).

**FIGURE 2:**
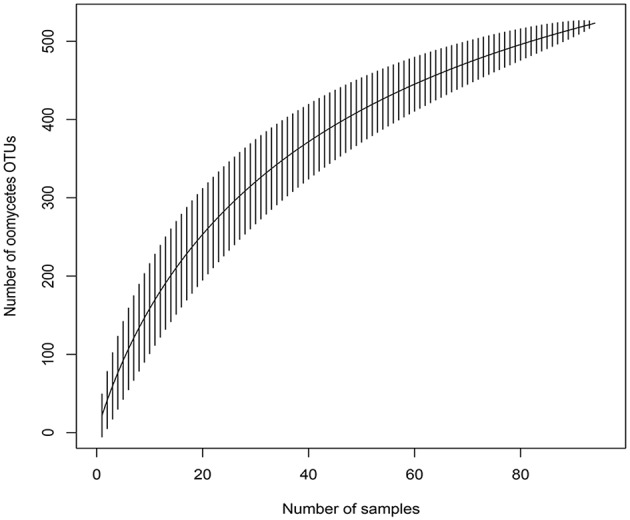
Species accumulation curve for the 96 samples, obtained with the function *specaccum* of the ‘vegan’ R package; it shows the relationship between observed OTUs and number of samples.

**FIGURE 3:**
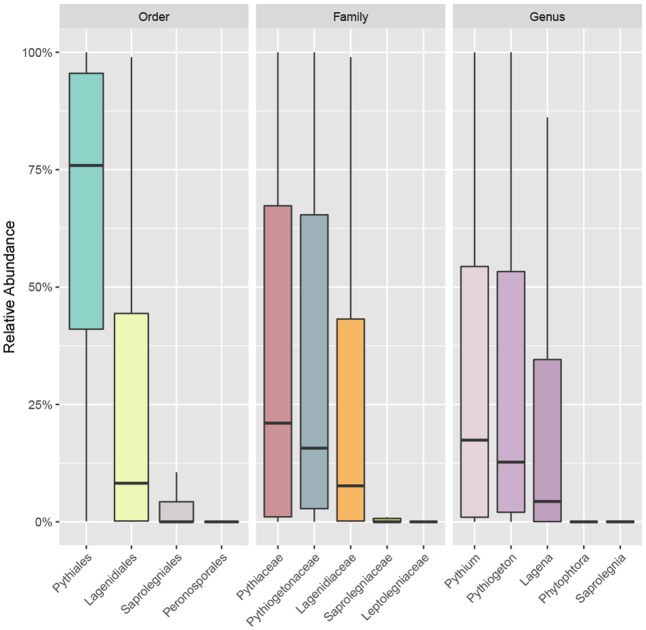
Boxplot showing the relative abundance of the most abundant taxa of oomycetes communities at order, family, and genus levels. Box borders represent the first and third quartiles, and central lines represent medians.

The accumulation curve showed that the number of OTUs hardly approached a plateau phase (**Figure 2**), indicating a higher oomycetes diversity in the area than was covered in this study and that diversity would increase with increasing sample number. In our dataset, Pythiales dominated the reads (66%), followed by Lagenidiales (25%), Saprolegniales (6.5%) and Peronosporales (2.4%), while at genus level we detected the presence of three dominating taxa: *Pythiogeton* (30.9%), *Pythium* (29.9%), and *Lagena* (22%) (**Figure 3**).

### Oomycete Diversity Analysis

**FIGURE 4:**
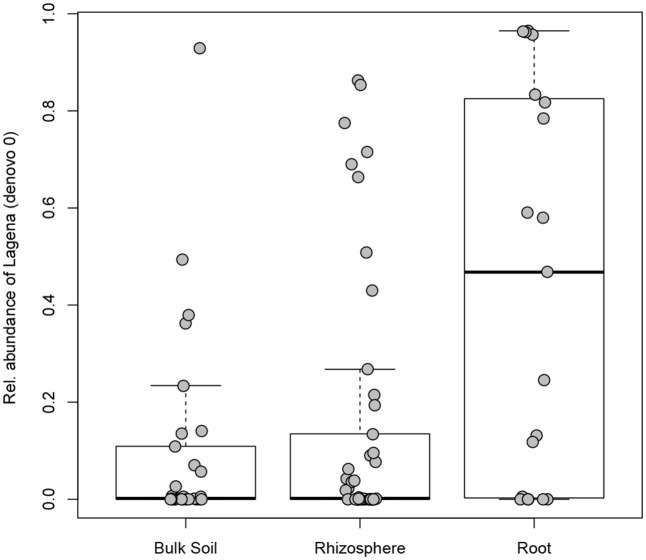
*Lagena* sp. distribution in bulk soil, rhizosphere, and root samples. Boxplot show the 25% and 75% quartiles of the data, the central line represents the median, bars extend to 95% confidence limits.

The incidence (the number of samples in which a certain OTU occurred) was checked for the 523 OTUs identified in this study: 166 OTUs were found in only one sample, while only one OTU (denovo0; *Lagena*) was found in more than 50% of the samples (see Supplementary Table S3). *Lagena* sp. (denovo0) had a significantly higher relative abundance in root samples in comparison to rhizosphere (Kruskal–Wallis test, *P* = 0.023) and bulk soil samples (Kruskal–Wallis test, *P* = 0.017) (**Figure 4**). The species richness was compared in relation to different sites, plant compartments (root, rhizosphere, or bulk soil), temporary or permanent flooding (i.e., absence or presence of die-back). Interestingly, none of the studied factors significantly influenced OTUs richness. The species evenness estimated by Pielou’s index, is significantly influenced by the compartment (df = 2; *P* = 0.017). Pairwise Wilcoxon rank sum tests revealed that the evenness is significantly different between root and bulk soil samples (*P* = 0.018).

### Factor Shaping Oomycete Communities

The effects of the geographical location, the compartment (bulk soil, rhizosphere soil, or root sample), die-back occurrence, and the available chemical data were subjected to permutation multivariate analysis of variance to evaluate factors that affected the structure (beta diversity) of oomycetes communities. The factors that were found to be significant in explaining the diversity were die-back occurrence (*R*^2^ = 0.049, *P* = 0.001), compartment (*R*^2^ = 0.043, *P* = 0.009), and the interaction between lake and die-back occurrence (*R*^2^ = 0.073, *P* = 0.011) (**Table 1**). Among the heavy metals, only cadmium concentration (mg⋅Kg^-1^) is significantly related to oomycetes distribution (*R*^2^ = 0.035, *P* = 0.002) (**Table 2**).

**Table 1:** Results from permutational multivariate analysis of variance to partition the variance in oomycete communities for the combined data. R_B_RT indicates the compartment: R, rhizosphere; B, bulk soil; RT, roots.

Variable	df	Sum sq	Mean sq	*F*-value	*R*^2^	Pr(>F)	
Lake	4	2.023	0.506	1.262	0.064	0.031	.
Die-back	1	1.553	1.553	3.874	0.049	0.001	^∗∗∗^
R_B_RT	2	1.368	0.683	1.776	0.043	0.009	^∗∗^
Lake:Die-back	4	2.308	0.577	1.499	0.073	0.011	^∗^
Lake: R_B_RT	8	3.347	0.418	1.087	0.106	0.216	
Die-back: R_B_RT	2	0.666	0.333	0.865	0.021	0.727	
Lake:Die-back:R_B_RT	5	2.099	0.419	1.091	0.067	0.258	
Residuals	47	18.091	0.385		0.575		

*^∗^p < 0.05; ^∗∗^p < 0.01; ^∗∗∗^p < 0.001.*

**Table 2:** Results from permutational multivariate analysis of variance to test the effect of heavy metals in the soils on oomycetes communities.

Variable	df	Sum sq	Mean sq	*F*-value	*R*^2^	Pr(>F)	
Cd	1	1.037	1.037	2.518	0.035	0.002	^∗∗^
Pb	1	0.593	0.593	1.440	0.020	0.093	.
Zn	1	0.600	0.600	1.457	0.020	0.079	.
Cr	1	0.600	0.600	1.457	0.020	0.085	.
Ni	1	0.551	0.551	1.338	0.019	0.144	
Cu	1	0.445	0.445	1.081	0.015	0.344	
Al	1	0.38	0.384	0.933	0.013	0.523	
Residuals	61	25.117	0.412		0.855		

*^∗^p < 0.05; ^∗∗^p < 0.01; ^∗∗∗^p < 0.001.*

**FIGURE 5:**
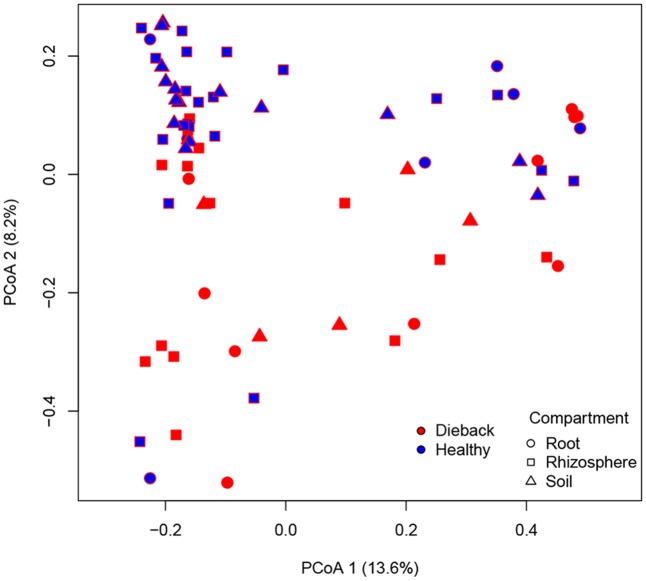
Principal coordinates analysis (PCoA) plot based on Bray–Curtis matrix. Samples are colored based on RDBS occurrence.

**FIGURE 6:**
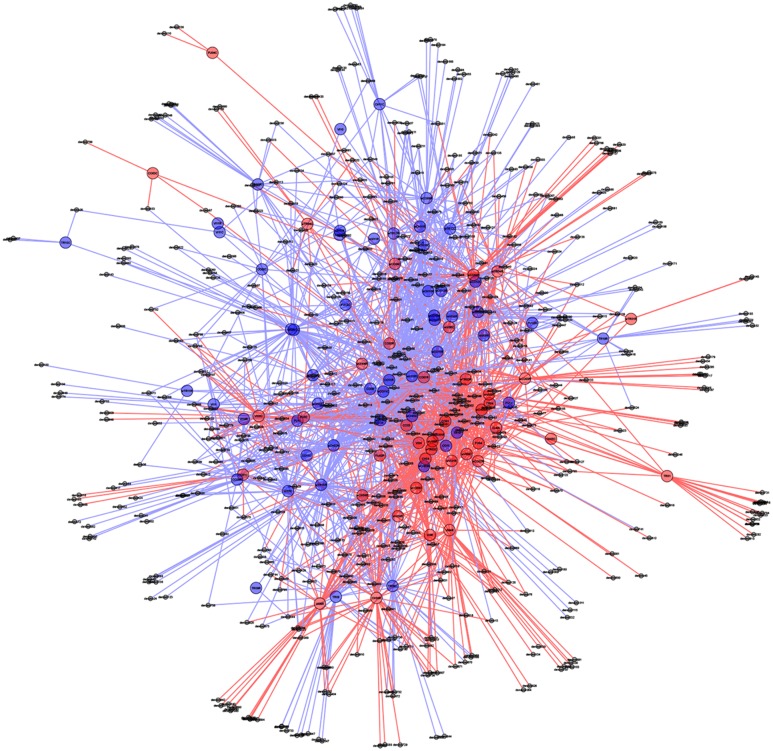
Oomycetes OTU networks in healthy and die-back samples. Larger nodes indicate samples, whereas smaller nodes represent OTUs. All OTUs are connected to the sample to which they belong with a line (edge) making shared OTUs connected to more than one sample. Healthy samples and OTUs as well as lines are indicated with blue, and die-back samples with red. Samples with shared OTUs are located in the center of the plot, while samples with unique OTUs are located in the periphery.

In support of this, a PCoA plot revealed clustering based on the plant health status, demonstrating the importance of die-back in explaining the diversity of oomycetes (**Figure 5**). The clustering of samples based on die-back was supported by covariance biplots (Supplementary Figure S1). The influence of die-back in oomycetes communities was also visible via network based analysis (**Figure 6**). The samples with higher number of shared OTUs were closer and clustered in the center of the network. The majority of die-back samples clustered in the lower core of the network. The network analysis also showed that a large part of OTUs were shared between healthy and RDBS samples.

### Difference between Healthy and RDBS Samples at OTU Level

Indicator species analysis of the total dataset revealed 12 OTUs that were associated with RDBS (**Table 3**).

**Table 3:** List of indicator species that were significantly different calculated using the Dufrene and Legendre analysis in R, considering the total dataset, only roots samples, and only rhizosphere samples.

OTU	BlastID	Health status	Indicator value	*P*-value
**Total dataset**				
denovo8	*Pythiogeton* sp.	Die-back	0.501	0.001
denovo1	*Pythiogeton* sp.	Die-back	0.413	0.004
denovo208	*Pythiogeton* sp.	Die-back	0.399	0.001
denovo3	*Pythiogeton* sp.	Die-back	0.340	0.002
denovo302	*Pythiogeton* sp.	Die-back	0.307	0.001
denovo34	*Pythiogeton* sp.	Die-back	0.302	0.001
denovo284	*Pythiogeton* sp.	Die-back	0.273	0.019
denovo594	*Pythiogeton* sp.	Die-back	0.253	0.003
denovo11	*Pythium intermedium*	Healthy	0.302	0.004
**Root**				
denovo8	*Pythiogeton* sp.	Die-back	0.690	0.014
denovo3	*Pythiogeton* sp.	Die-back	0.689	0.029
denovo152	*Pythiogeton* sp.	Die-back	0.500	0.027
denovo245	*Pythiogeton* sp.	Die-back	0.500	0.038
**Rhizosphere**				
denovo8	*Pythiogeton* sp.	Die-back	0.438	0.023
denovo208	*Pythiogeton* sp.	Die-back	0.360	0.011
denovo302	*Pythiogeton* sp.	Die-back	0.331	0.026
denovo34	*Pythiogeton* sp.	Die-back	0.327	0.009
denovo3	*Pythiogeton* sp.	Die-back	0.299	0.038
denovo1113	*Pythiogeton* sp.	Die-back	0.265	0.049
denovo436	*Pythiogeton* sp.	Die-back	0.262	0.050
denovo245	*Pythiogeton* sp.	Die-back	0.250	0.010
denovo10	*Pythiogeton* sp.	Healthy	0.280	0.046
denovo11	*Pythium intermedium*	Healthy	0.280	0.038

**FIGURE 7:**
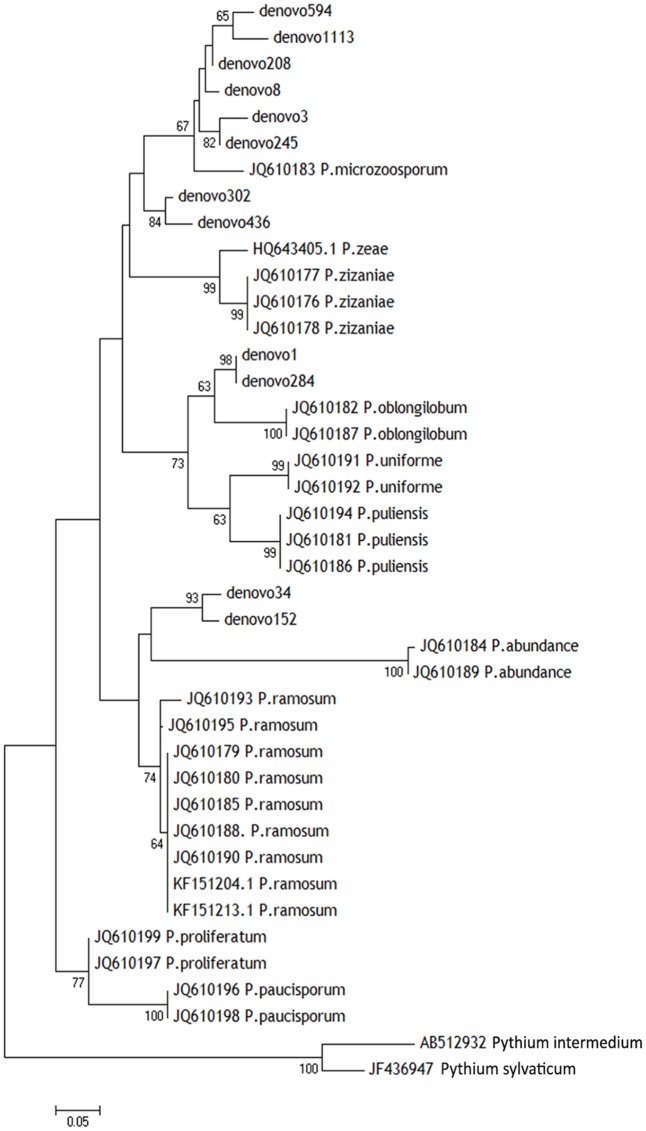
Neighbor-joining phylogenetic tree based on ITS1 region showing the diversity of *Pythiogeton* species in our root, rhizosphere, and bulk soil samples. Sequences from this study are shown with *denovo* numbers, whereas reference sequences are listed with the NCBI accession numbers. The two most abundant *Pythium* species were used as outgroups. Numbers at branching points are bootstrap percentages based on 1000 replications. Only values > 50% are shown.

Those 12 OTUs belonged to the genus *Pythiogeton.* The relative abundance of *Pythiogeton* was significantly higher in die-back samples in comparison to healthy (*P* = 0.0002) (Supplementary Figure S2). A phylogenetic tree (**Figure 7**) shows that eight of the OTUs (denovo594, denovo1113, denovo208, denovo8, denovo3, denovo245, denovo302, denovo246) are similar to *Pythiogeton microzoosporum*, although the clades do not show a high bootstrap support. Denovo1 and denovo284 are similar (bootstrap = 98) and close to the clade containing *Pythiogeton oblongilobum* (bootstrap = 63). Denovo34 and denovo152 cluster together (bootstrap = 100) and they are similar to *Pythiogeton abundance*.

## Discussion

Die-back syndrome is a complex phenomenon affecting mature stands of *P. australis*. It represents a serious threat to reed-dominated ecosystems, given the importance of the plant in protecting the shoreline from bank erosion and in providing food resource or protection for arthropods, birds and mammals (Ostendorp et al., 2003). Even though different abiotic factors (for example, permanent flooding of the stand and presence of heavy metals) have been suggested as possible causes of this syndrome (Gigante et al., 2014; Lastrucci et al., 2016a), a clear understanding of this phenomenon is still missing. The role of biotic factor is also poorly understood; in particular, the interaction between plant and microorganisms is not completely explored yet. To this aim, the present study analyzed the distribution of oomycetes communities in five freshwater ecosystems and investigated a possible correlation with the health status of *P. australis*.

As reported by Wielgoss et al. (2009), the oomycete *P. phragmitis* was considered to be often associated with reed die-back in Lake Constance, Germany, especially if co-occurring with permanent flooding condition of the plant. Interestingly, we did not detect *P. phragmitis*, in any of our 96 samples. We attempted a qPCR approach, using the primers described by Wielgoss et al. (2009) for the detection of *P. phragmitis*, however, with no success (data not shown). The reason for the absence of *P. phragmitis* in samples from Italy could be the different bioclimatic conditions between Germany and Italy.

In our study, instead, indicator species analysis revealed 12 OTUs which showed significant differences in abundance between stands where *P. australis* is affected by die-back syndrome and stands in good conservation status. All the OTUs identified as indicator species in RDBS belong to the genus *Pythiogeton*. *Pythiogeton* is a poorly studied oomycetes genus containing 16 known species (MycoBank^3^). It is difficult to study because of its reduced capacity to grow in culture; indeed, it was believed to be a saprophyte until recent investigations proved its pathogenicity, also toward Poaceae members (Doan et al., 2013; Huang et al., 2013). As already demonstrated (Natvig and Gleason, 1983; Huang et al., 2013), *Pythiogeton* species are more virulent in anaerobic condition, and this could be the reason why they were detected in die-back stands. In fact, reed die-back, as showed in several former studies (Gigante et al., 2011, 2014; Lastrucci et al., 2016a) and even confirmed in the presently considered areas (Lastrucci et al., 2016b), mostly affects stands with prolonged or even permanent conditions of submersions. Indeed, in the study areas the stands affected by RDBS (group Y) are permanently flooded, with water persistence even in the driest period (i.e., the end of summer), while those in a good health status (group N) are only temporarily flooded, emerging at least at the end of summer. A phylogenetic tree was constructed to understand the relationship between the known species of *Pythiogeton* and the OTUs assembled in this study. Eight of them are closely related to *P. microzoosporum*, two OTUs were similar to the branch with *P. oblongilobum*, and two were close to *P. abundance*. *P. oblongilobum* was isolated by Huang et al. (2013) in the stem debris of the water bamboo (*Zizania latifolia*). *P. abundance* was firstly isolated from decaying leaves of common cattail (Drechsler, 1932), and later by Huang et al. (2013). In previous investigations (Xu et al., 2012; Nelson and Karp, 2013; Sapkota and Nicolaisen, 2015) *Pythiogeton* was not detected at all, or only at low levels; instead, in our work, it was the most abundant genus, together with *Pyhtium*. One reason can be in the different approaches used: Nelson and Karp (2013) used the cloning, Xu et al. (2012) used different primer pairs to include both fungi and oomycetes, while we used primers specific for oomycetes. Another obvious reason can be in the different considered ecosystem, as Sapkota and Nicolaisen (2015) worked on carrot fields in Denmark, while we work on freshwater ecosystems. At last, the co-occurrence of *Pythiogeton* with RDBS could also be the reason of the findings.

In the PCoA plot, most of the samples from healthy stands clustered together, while RDBS samples were spread in the plot. Nevertheless, the network analysis indicated that a large part of the OTUs are shared between the two categories, healthy or die-back affected samples, and the main differences between them were the differences in *Pythiogeton* spp. distribution. These results supported a possible relation between die-back occurrence and oomycetes communities composition.

Interestingly, we found that OTUs richness is not statistically different among the considered lakes, nor between compartments or health status of the plant. Species evenness, on the other hand, was significantly affected by the compartment (root, rhizosphere, bulk soil). This means that species are not evenly distributed and their relative abundance change based on the sample type. *Lagena* sp. was indeed more widely present in the roots samples, compared to rhizosphere and bulk soil; it is an obligate intercellular parasite of plant roots (Barr and Dèsaulniers, 1990), observed also in wild grasses (Blackwell, 2011; Bakker et al., 2017), but little investigation has been done to elucidate its taxonomy and pathogenicity because of difficulties in cultivation. In this study, hardly any differences in the relative abundance of *Lagena* were detected between healthy and declining stands, indicating that it cannot be related to RDBS development in our samples.

In this study, we also took the chemistry of the soils into account, as previous investigations highlighted a possible role of heavy metals in oomycetes growth (Henderson et al., 2017). Among the heavy metals analyzed, the concentration of cadmium was the only one that showed a significant relationship with oomycete distribution. The toxicity of cadmium for soil microbial biomass growth and metabolic activity is well-known (Xu et al., 2013) and Cd^2+^ concentrations has been demonstrated to affect soil microbial diversity (Chien et al., 2008; Ribeiro et al., 2016). Several studies have also demonstrated the effect of Cadmium on *P. australis* fitness (Ederli et al., 2004) and in RDBS (Lastrucci et al., 2016a).

To summarize, RDBS remains a very intricate phenomenon, which is the result of the interaction between abiotic and biotic factors. Stress conditions determined by flooding and heavy metals could in fact be worsened by oomycetes presence. This is the first metabarcoding study of oomycete diversity in reed beds ecosystems. Our approach allowed to highlight results that would have been missed with cultivation-based studies, and demonstrated the presence of *Pythiogeton* as associated to declining reed stands, which occur to be permanently flooded. Hopefully, these new insights will contribute to unravel the diversity of these poorly studied organisms living in reed-dominated ecosystems.

## Author Contributions

MC, MN, LR, and FF designed the work; MC, VF, DG, AC, and LL collected the samples; MC, RSe, and RSa acquired and analyzed the data; MC drafted the manuscript; RSe, MN, DG, LR, AC, LL, FF, RV, BF, RSa critically revised the article. All the authors approved the version of the manuscript to be published.

## Conflict of Interest Statement

The authors declare that the research was conducted in the absence of any commercial or financial relationships that could be construed as a potential conflict of interest.

## Footnotes

1 http://www.ncbi.nlm.nih.gov/sra

2 http://cytoscape.org/

3 http://www.mycobank.org/
