# Supplementary material for: Oomycete Communities Associated with Reed Die-Back Syndrome
Source: Front Plant Sci. 2017 Sep 7;8:1550. doi: 10.3389/fpls.2017.01550 (PMC5594075; doi:10.3389/fpls.2017.01550)
Supplement: Supplementary file 1 [file Table_1.docx]

**Table S1**: Summary of the samples used in this study and their main characteristics.

| SampleID | MID sequence | Lake | Sampling time | R_B_RT* | Die-back** | P_T*** | Cd  (mg·Kg^-1^) | Pb (mg·Kg^-1^) | Zn  (mg·Kg^-1^) | Cr  (mg·Kg^-1^) | Ni  (mg·Kg^-1^) | Cu  (mg·Kg^-1^) | Al  (mg·Kg^-1^) | Latitude | Longitude |
| --- | --- | --- | --- | --- | --- | --- | --- | --- | --- | --- | --- | --- | --- | --- | --- |
| CH01C | ACGAGTGCGT | Chiusi | Sept. 2015 | B | N | P | 5.499 | 50.498 | 119.555 | 134.082 | 146.792 | 58.294 | 45512.381 | 43.05402 | 11.95052 |
| CH01R | ACGCTCGACA | Chiusi | Sept. 2015 | R | N | P | 5.499 | 50.498 | 119.555 | 134.082 | 146.792 | 58.294 | 45512.381 | 43.05402 | 11.95052 |
| CH04 | AGACGCACTC | Chiusi | Sept. 2015 | RT | Y | P | 0.699 | 2.320 | 49.926 | 5.177 | 7.695 | 6.655 | 6523.328 | 43.05345 | 11.97723 |
| CH04R | ATCAGACACG | Chiusi | Sept. 2015 | R | Y | P | 6.556 | 17.419 | 111.186 | 141.657 | 104.303 | 35.742 | 58408.466 | 43.05345 | 11.97723 |
| CH05 | AGCACTGTAG | Chiusi | Sept. 2015 | RT | Y | P | 0.371 | 1.445 | 40.050 | 5.117 | 5.685 | 8.059 | 2328.645 | 43.05568 | 11.97765 |
| CH05C | ATATCGCGAG | Chiusi | Sept. 2015 | B | Y | P | 6.725 | 23.566 | 133.245 | 175.006 | 131.669 | 44.293 | 56810.071 | 43.05568 | 11.97765 |
| CH11 | CGTGTCTCTA | Chiusi | Sept. 2015 | RT | N | T | 1.266 | 1.952 | 35.065 | 7.480 | 8.832 | 11.237 | 7282.524 | 43.0567 | 11.97897 |
| CH11C | CTCGCGTGTC | Chiusi | Sept. 2015 | B | N | T | 10.160 | 10.261 | 141.483 | 162.461 | 120.812 | 29.464 | 55032.239 | 43.0567 | 11.97897 |
| CH11R | TCTCTATGCG | Chiusi | Sept. 2015 | R | N | T | 10.160 | 10.261 | 141.483 | 162.461 | 120.812 | 29.464 | 55032.239 | 43.0567 | 11.97897 |
| CO01 | ACGACTACAG | Colfiorito | Sept. 2015 | RT | Y | P | 1.025 | 0.382 | 71.448 | 0.286 | 4.011 | 2.903 | 5239.228 | 43.02663 | 12.87625 |
| CO01C | TGATACGTCT | Colfiorito | Sept. 2015 | B | Y | P | 2.463 | 9.756 | 73.545 | 24.381 | 27.937 | 22.832 | 15263.747 | 43.02663 | 12.87625 |
| CO01R | CATAGTAGTG | Colfiorito | Sept. 2015 | R | Y | P | 2.463 | 9.756 | 73.545 | 24.381 | 27.937 | 22.832 | 15263.747 | 43.02827 | 12.87633 |
| CO03 | TACGAGTATG | Colfiorito | Sept. 2015 | RT | Y | P | 0.774 | 1.810 | 90.237 | 4.039 | 14.799 | 9.566 | 3737.625 | 43.02827 | 12.87633 |
| CO03C | CGAGAGATAC | Colfiorito | Sept. 2015 | B | Y | P | 3.767 | 9.059 | 78.269 | 43.933 | 47.286 | 19.068 | 21140.473 | 43.02827 | 12.87633 |
| CO03R | ATACGACGTA | Colfiorito | Sept. 2015 | R | Y | P | 3.767 | 9.059 | 78.269 | 43.933 | 47.286 | 19.068 | 21140.473 | 43.02605 | 12.87915 |
| CO09 | TAGAGACGAG | Colfiorito | Sept. 2015 | RT | N | T | 2.727 | 3.533 | 43.683 | 5.400 | 8.579 | 11.140 | 9375.998 | 43.02605 | 12.87915 |
| CO09C | TCACGTACTA | Colfiorito | Sept. 2015 | B | N | T | 7.350 | 18.479 | 124.994 | 58.880 | 83.894 | 37.570 | 39745.960 | 43.02605 | 12.87915 |
| CO09R | CGTCTAGTAC | Colfiorito | Sept. 2015 | R | N | T | 7.350 | 18.479 | 124.994 | 58.880 | 83.894 | 37.570 | 39745.960 | 43.02362 | 12.86937 |
| CO11 | TCGTCGCTCG | Colfiorito | Sept. 2015 | RT | N | T | 0.881 | 2.075 | 34.755 | 2.242 | 9.763 | 6.724 | 2489.871 | 43.02362 | 12.86937 |
| CO11C | TCTACGTAGC | Colfiorito | Sept. 2015 | B | N | T | 3.334 | 9.459 | 49.191 | 14.663 | 23.634 | 14.605 | 14795.639 | 43.02362 | 12.86937 |
| CO11R | TGTACTACTC | Colfiorito | Sept. 2015 | R | N | T | 3.334 | 9.459 | 49.191 | 14.663 | 23.634 | 14.605 | 14795.639 | 43.02663 | 12.87625 |
| FU02 | ACTACTATGT | Fucecchio | Sept. 2015 | RT | Y | P | NA | 6.560 | 30.389 | NA | 7.052 | 5.534 | 14242.020 | 43.80577 | 10.8062 |
| FU02R | CGTAGACTAG | Fucecchio | Sept. 2015 | R | Y | P | 6.485 | 139.462 | 139.144 | 136.930 | 92.545 | 36.759 | 41505.636 | 43.80577 | 10.8062 |
| FU04 | CAGTAGACGT | Fucecchio | Sept. 2015 | RT | Y | P | NA | 18.482 | 33.241 | 6.961 | 10.820 | 9.967 | 7773.007 | 43.80412 | 10.8012 |
| FU04C | TACTCTCGTG | Fucecchio | Sept. 2015 | B | Y | P | 6.435 | 81.226 | 174.483 | 130.780 | 101.209 | 52.936 | 39367.152 | 43.80412 | 10.8012 |
| FU10 | TAGTGTAGAT | Fucecchio | Sept. 2015 | RT | N | T | 1.487 | 18.172 | 67.985 | 23.931 | 31.480 | 33.941 | 6043.606 | 43.81148 | 10.81172 |
| FU10R | ACATACGCGT | Fucecchio | Sept. 2015 | R | N | T | 8.936 | 28.136 | 162.164 | 148.817 | 111.601 | 39.477 | 67120.379 | 43.81148 | 10.81172 |
| FU12 | TCGATCACGT | Fucecchio | Sept. 2015 | RT | N | T | 1.221 | 23.165 | 89.487 | 8.261 | 13.339 | 43.073 | 5749.427 | 43.81297 | 10.81045 |
| FU12C | ACGCGAGTAT | Fucecchio | Sept. 2015 | B | N | T | 9.992 | 47.184 | 138.389 | 167.326 | 124.154 | 42.851 | 73798.255 | 43.81297 | 10.81045 |
| TR01 | TCGCACTAGT | Trasimeno | Sept. 2015 | RT | Y | P | 0.902 | 0.221 | 97.223 | 0.224 | NA | 2.983 | 390.963 | 43.09202 | 12.17797 |
| TR04 | TCTAGCGACT | Trasimeno | Sept. 2015 | RT | Y | P | 1.031 | 1.957 | 65.178 | 2.483 | NA | NA | 570.889 | 43.10067 | 12.1866 |
| TR09 | TCTATACTAT | Trasimeno | Sept. 2015 | RT | N | T | 0.374 | 1.049 | 37.844 | 5.073 | 5.392 | 19.500 | 2355.260 | 43.09775 | 12.0673 |
| TR09C | ACTGTACAGT | Trasimeno | Sept. 2015 | B | N | T | 4.084 | 0.657 | 25.274 | 33.067 | 19.699 | 6.548 | 17837.357 | 43.09775 | 12.0673 |
| TR09R | AGACTATACT | Trasimeno | Sept. 2015 | R | N | T | 4.084 | 0.657 | 25.274 | 33.067 | 19.699 | 6.548 | 17837.357 | 43.09775 | 12.0673 |
| TR10 | TGACGTATGT | Trasimeno | Sept. 2015 | RT | N | T | 1.048 | 4.178 | 44.204 | 3.522 | 6.460 | 7.871 | 9035.613 | 43.08962 | 12.08182 |
| TR10C | AGCGTCGTCT | Trasimeno | Sept. 2015 | B | N | T | 3.392 | 1.062 | 19.926 | 17.997 | 18.354 | 6.532 | 14183.789 | 43.08962 | 12.08182 |
| TR10R | AGTACGCTAT | Trasimeno | Sept. 2015 | R | N | T | 3.392 | 1.062 | 19.926 | 17.997 | 18.354 | 6.532 | 14183.789 | 43.08962 | 12.08182 |
| VI04 | TGTGAGTAGT | Vico | Sept. 2015 | RT | Y | P | 1.099 | 3.937 | 74.161 | 0.691 | 4.213 | 5.928 | 8789.666 | 42.32455 | 12.19692 |
| VI04C | ATAGAGTACT | Vico | Sept. 2015 | B | Y | P | 1.627 | 26.318 | 59.677 | 9.861 | 20.499 | 24.079 | 19353.354 | 42.32455 | 12.19692 |
| VI04R | CACGCTACGT | Vico | Sept. 2015 | R | Y | P | 1.627 | 26.318 | 59.677 | 9.861 | 20.499 | 24.079 | 19353.354 | 42.32455 | 12.19692 |
| VI06 | ACAGTATATA | Vico | Sept. 2015 | RT | Y | P | 3.114 | 4.043 | 57.161 | 0.831 | 16.392 | 5.282 | 22243.286 | 42.33947 | 12.15747 |
| VI06R | CGACGTGACT | Vico | Sept. 2015 | R | Y | T | 3.824 | 33.874 | 45.947 | 13.492 | 21.651 | 18.900 | 39394.951 | 42.33947 | 12.15747 |
| VI10 | ACGCGATCGA | Vico | Sept. 2015 | RT | N | T | 2.791 | 8.882 | 77.484 | 0.287 | 8.062 | 8.201 | 14694.380 | 42.33302 | 12.15375 |
| VI10C | TACACACACT | Vico | Sept. 2015 | B | N | T | 4.509 | 10.444 | 38.845 | 2.674 | 11.724 | 8.032 | 20611.496 | 42.33302 | 12.15375 |
| VI10R | TACACGTGAT | Vico | Sept. 2015 | R | N | T | 4.509 | 10.444 | 38.845 | 2.674 | 11.724 | 8.032 | 20611.496 | 42.33302 | 12.15375 |
| VI11 | ACTAGCAGTA | Vico | Sept. 2015 | RT | N | T | 4.470 | 9.017 | 67.261 | 2.361 | 18.999 | 2.056 | 60648.696 | 42.3337 | 12.15227 |
| VI11C | TACAGATCGT | Vico | Sept. 2015 | B | N | T | 5.944 | 45.483 | 46.021 | 12.486 | 16.492 | 14.598 | 31721.745 | 42.3337 | 12.15227 |
| VI11R | TACGCTGTCT | Vico | Sept. 2015 | R | N | T | 5.944 | 45.483 | 46.021 | 12.486 | 16.492 | 14.598 | 31721.745 | 42.3337 | 12.15227 |
| wCH01R | ACGAGTGCGT | Chiusi | Febr. 2015 | R | N | F | 5.499 | 50.498 | 119.555 | 134.082 | 146.792 | 58.294 | 45512.381 | 43.05402 | 11.95052 |
| wCH02C | TCTCTATGCG | Chiusi | Febr. 2015 | B | N | F | 6.915 | 35.338 | 116.555 | 128.072 | 125.011 | 52.402 | 58300.164 | 43.0555 | 11.9495 |
| wCH02R | ACGCTCGACA | Chiusi | Febr. 2015 | R | N | F | 6.915 | 35.338 | 116.555 | 128.072 | 125.011 | 52.402 | 58300.164 | 43.0555 | 11.9495 |
| wCH03C | TGATACGTCT | Chiusi | Febr. 2015 | B | N | F | 2.945 | 25.649 | 95.771 | 79.960 | 78.855 | 38.328 | 27195.771 | 43.05668 | 11.95037 |
| wCH03R | AGACGCACTC | Chiusi | Febr. 2015 | R | N | F | 2.945 | 25.649 | 95.771 | 79.960 | 78.855 | 38.328 | 27195.771 | 43.05668 | 11.95037 |
| wCH04R | AGCACTGTAG | Chiusi | Febr. 2015 | R | Y | P | 6.556 | 17.419 | 111.186 | 141.657 | 104.303 | 35.742 | 58408.466 | 43.05345 | 11.97723 |
| wCH05R | ATCAGACACG | Chiusi | Febr. 2015 | R | Y | P | 6.725 | 23.566 | 133.245 | 175.006 | 131.669 | 44.293 | 56810.071 | 43.05568 | 11.97765 |
| wCH06R | ATATCGCGAG | Chiusi | Febr. 2015 | R | Y | P | 6.149 | 19.736 | 118.927 | 151.470 | 116.185 | 43.785 | 55308.280 | 43.05668 | 11.97783 |
| wCH11C | CATAGTAGTG | Chiusi | Febr. 2015 | B | N | T | 10.160 | 10.261 | 141.483 | 162.461 | 120.812 | 29.464 | 55032.239 | 43.0567 | 11.97897 |
| wCH11R | CGTGTCTCTA | Chiusi | Febr. 2015 | R | N | T | 10.160 | 10.261 | 141.483 | 162.461 | 120.812 | 29.464 | 55032.239 | 43.0567 | 11.97897 |
| wCH12C | CGAGAGATAC | Chiusi | Febr. 2015 | B | N | T | 9.935 | 50.157 | 137.163 | 152.416 | 101.922 | 82.148 | 64790.557 | 43.05482 | 11.94672 |
| wCH12R | CTCGCGTGTC | Chiusi | Febr. 2015 | R | N | T | 9.935 | 50.157 | 137.163 | 152.416 | 101.922 | 82.148 | 64790.557 | 43.05482 | 11.94672 |
| wCO01R | ATACGACGTA | Colfiorito | Febr. 2015 | R | Y | P | 2.463 | 9.756 | 73.545 | 24.381 | 27.937 | 22.832 | 15263.747 | 43.02663 | 12.87625 |
| wCO02R | TCACGTACTA | Colfiorito | Febr. 2015 | R | Y | P | 2.572 | 47.281 | 71.218 | 17.441 | 22.480 | 22.353 | 13309.062 | 43.02517 | 12.87553 |
| wCO03C | TACTCTCGTG | Colfiorito | Febr. 2015 | B | Y | P | 3.767 | 9.059 | 78.269 | 43.933 | 47.286 | 19.068 | 21140.473 | 43.02827 | 12.87633 |
| wCO03R | CGTCTAGTAC | Colfiorito | Febr. 2015 | R | Y | P | 3.767 | 9.059 | 78.269 | 43.933 | 47.286 | 19.068 | 21140.473 | 43.02827 | 12.87633 |
| wCO04C | TAGAGACGAG | Colfiorito | Febr. 2015 | B | Y | P | 3.810 | 22.425 | 89.446 | 47.187 | 51.878 | 24.162 | 19080.402 | 43.02757 | 12.87728 |
| wCO04R | TCTACGTAGC | Colfiorito | Febr. 2015 | R | Y | P | 3.810 | 22.425 | 89.446 | 47.187 | 51.878 | 24.162 | 19080.402 | 43.02757 | 12.87728 |
| wCO09R | TGTACTACTC | Colfiorito | Febr. 2015 | R | N | T | 7.350 | 18.479 | 124.994 | 58.880 | 83.894 | 37.570 | 39745.960 | 43.02605 | 12.87915 |
| wCO10R | ACGACTACAG | Colfiorito | Febr. 2015 | R | N | T | 4.338 | 9.579 | 56.254 | 28.270 | 33.130 | 19.843 | 23796.990 | 43.02035 | 12.87887 |
| wCO11C | TCGTCGCTCG | Colfiorito | Febr. 2015 | B | N | T | 3.334 | 9.459 | 49.191 | 14.663 | 23.634 | 14.605 | 14795.639 | 43.02362 | 12.86937 |
| wCO11R | CGTAGACTAG | Colfiorito | Febr. 2015 | R | N | T | 3.334 | 9.459 | 49.191 | 14.663 | 23.634 | 14.605 | 14795.639 | 43.02362 | 12.86937 |
| wCO12C | ACATACGCGT | Colfiorito | Febr. 2015 | B | N | T | 5.037 | 16.403 | 91.500 | 37.147 | 43.792 | 25.033 | 30780.166 | 43.01878 | 12.87275 |
| wCO12R | TACGAGTATG | Colfiorito | Febr. 2015 | R | N | T | 5.037 | 16.403 | 91.500 | 37.147 | 43.792 | 25.033 | 30780.166 | 43.01878 | 12.87275 |
| wTR01R | ACGCGAGTAT | Trasimeno | Febr. 2015 | R | Y | P | 3.980 | 29.945 | 101.406 | 88.837 | 81.813 | 40.360 | 40626.496 | 43.09202 | 12.17797 |
| wTR02C | CAGTAGACGT | Trasimeno | Febr. 2015 | B | Y | P | 4.134 | 26.163 | 91.335 | 75.205 | 62.996 | 43.040 | 47269.531 | 43.09485 | 12.18478 |
| wTR02R | ACTACTATGT | Trasimeno | Febr. 2015 | R | Y | P | 4.134 | 26.163 | 91.335 | 75.205 | 62.996 | 43.040 | 47269.531 | 43.09485 | 12.18478 |
| wTR03R | ACTGTACAGT | Trasimeno | Febr. 2015 | R | Y | P | 4.149 | 30.505 | 82.490 | 91.187 | 69.782 | 46.819 | 43657.572 | 43.10282 | 12.18618 |
| wTR04C | CGACGTGACT | Trasimeno | Febr. 2015 | B | Y | P | 3.997 | 41.255 | 96.901 | 95.430 | 75.762 | 43.220 | 42752.499 | 43.10067 | 12.1866 |
| wTR04R | AGACTATACT | Trasimeno | Febr. 2015 | R | Y | P | 3.997 | 41.255 | 96.901 | 95.430 | 75.762 | 43.220 | 42752.499 | 43.10067 | 12.1866 |
| wTR09R | AGCGTCGTCT | Trasimeno | Febr. 2015 | R | N | T | 4.084 | 0.657 | 25.274 | 33.067 | 19.699 | 6.548 | 17837.357 | 43.09775 | 12.0673 |
| wTR10C | TACACACACT | Trasimeno | Febr. 2015 | B | N | T | 3.392 | 1.062 | 19.926 | 17.997 | 18.354 | 6.532 | 14183.789 | 43.08962 | 12.08182 |
| wTR10R | AGTACGCTAT | Trasimeno | Febr. 2015 | R | N | T | 3.392 | 1.062 | 19.926 | 17.997 | 18.354 | 6.532 | 14183.789 | 43.08962 | 12.08182 |
| wTR11R | ATAGAGTACT | Trasimeno | Febr. 2015 | R | N | T | 3.322 | NA | 23.476 | 27.395 | 10.042 | 4.743 | 12053.389 | 43.08223 | 12.10245 |
| wTR12C | TACACGTGAT | Trasimeno | Febr. 2015 | B | N | T | 5.587 | 16.083 | 62.978 | 70.935 | 53.922 | 12.794 | 30352.028 | 43.1911 | 12.1083 |
| wTR12R | CACGCTACGT | Trasimeno | Febr. 2015 | R | N | T | 5.587 | 16.083 | 62.978 | 70.935 | 53.922 | 12.794 | 30352.028 | 43.1911 | 12.1083 |
| wVI01R | TACAGATCGT | Vico | Febr. 2015 | R | Y | P | 1.484 | 22.363 | 56.834 | 10.258 | 15.555 | 17.762 | 18876.595 | 42.31915 | 12.19562 |
| wVI03R | TACGCTGTCT | Vico | Febr. 2015 | R | Y | P | 2.305 | 31.258 | 68.971 | 15.535 | 21.598 | 29.091 | 23624.273 | 42.32158 | 12.1948 |
| wVI04C | TGTGAGTAGT | Vico | Febr. 2015 | B | Y | P | 1.627 | 26.318 | 59.677 | 9.861 | 20.499 | 24.079 | 19353.354 | 42.32395 | 12.19692 |
| wVI04R | TAGTGTAGAT | Vico | Febr. 2015 | R | Y | P | 1.627 | 26.318 | 59.677 | 9.861 | 20.499 | 24.079 | 19353.354 | 42.32395 | 12.19692 |
| wVI06C | ACAGTATATA | Vico | Febr. 2015 | B | Y | P | 3.824 | 33.874 | 45.947 | 13.492 | 21.651 | 18.900 | 39394.951 | 42.33947 | 12.15747 |
| wVI06R | TCGATCACGT | Vico | Febr. 2015 | R | Y | P | 3.824 | 33.874 | 45.947 | 13.492 | 21.651 | 18.900 | 39394.951 | 42.33947 | 12.15747 |
| wVI09R | TCGCACTAGT | Vico | Febr. 2015 | R | N | T | 9.259 | 45.367 | 80.621 | 25.611 | 26.681 | 29.662 | 57193.459 | 42.33997 | 12.16287 |
| wVI10R | TCTAGCGACT | Vico | Febr. 2015 | R | N | T | 4.509 | 10.444 | 38.845 | 2.674 | 11.724 | 8.032 | 20611.496 | 42.33302 | 12.15375 |
| wVI11C | ACGCGATCGA | Vico | Febr. 2015 | B | N | T | 5.944 | 45.483 | 46.021 | 12.486 | 16.492 | 14.598 | 31721.745 | 42.3337 | 12.15227 |
| wVI11R | TCTATACTAT | Vico | Febr. 2015 | R | N | T | 5.944 | 45.483 | 46.021 | 12.486 | 16.492 | 14.598 | 31721.745 | 42.3337 | 12.15227 |
| wVI16C | ACTAGCAGTA | Vico | Febr. 2015 | B | N | T | 9.606 | 44.756 | 65.125 | 22.851 | 29.176 | 25.553 | 65956.386 | 42.33982 | 12.16213 |
| wVI16R | TGACGTATGT | Vico | Febr. 2015 | R | N | T | 9.606 | 44.756 | 65.125 | 22.851 | 29.176 | 25.553 | 65956.386 | 42.33982 | 12.16213 |

*R= rhizosphere; B=bulk soil; RT= roots; **N=no; Y=yes ***P=permanent flooding; T=temporary flooding.
