# Supplementary material for: Oomycete Communities Associated with Reed Die-Back Syndrome
Source: Front Plant Sci. 2017 Sep 7;8:1550. doi: 10.3389/fpls.2017.01550 (PMC5594075; doi:10.3389/fpls.2017.01550)
Supplement: Supplementary file 2 [file Table_2.DOCX]

| Analyte | Wavelength  (nm) | Detection limits sediments  (mg·kg^−1^) | Detection limits plant tissues (mg·kg^−1^) |
| --- | --- | --- | --- |
| Cd | 214.438 | 0.510 | 0.137 |
| Pb | 220.353 | 0.356 | 0.095 |
| Zn | 213.856 | 0.389 | 0.104 |
| Cr | 267.716 | 0.380 | O.102 |
| Ni | 216.556 | 0.469 | 0.126 |
| Cu | 327.396 | 0.538 | 0.144 |
| Al | 237.312 | 1.517 | 0.407 |

**Table S2**:Wavelengths and estimated detection limits for chemical analysis.
