# Supplementary figures and images for: Oomycete Communities Associated with Reed Die-Back Syndrome
Source: Front Plant Sci. 2017 Sep 7;8:1550. doi: 10.3389/fpls.2017.01550 (PMC5594075; doi:10.3389/fpls.2017.01550)

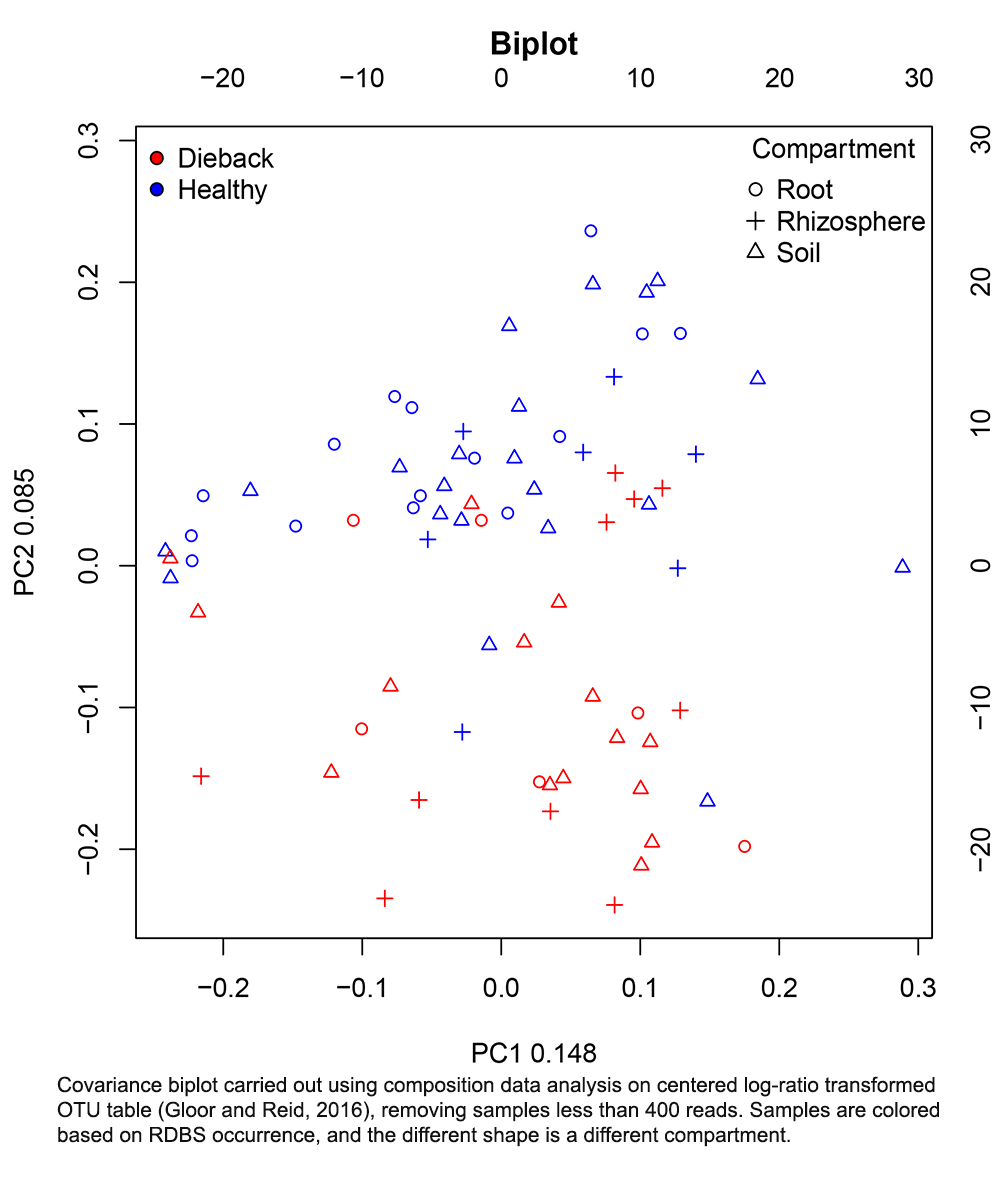

Supplement: Supplementary file 4 [file Image_1.TIF]

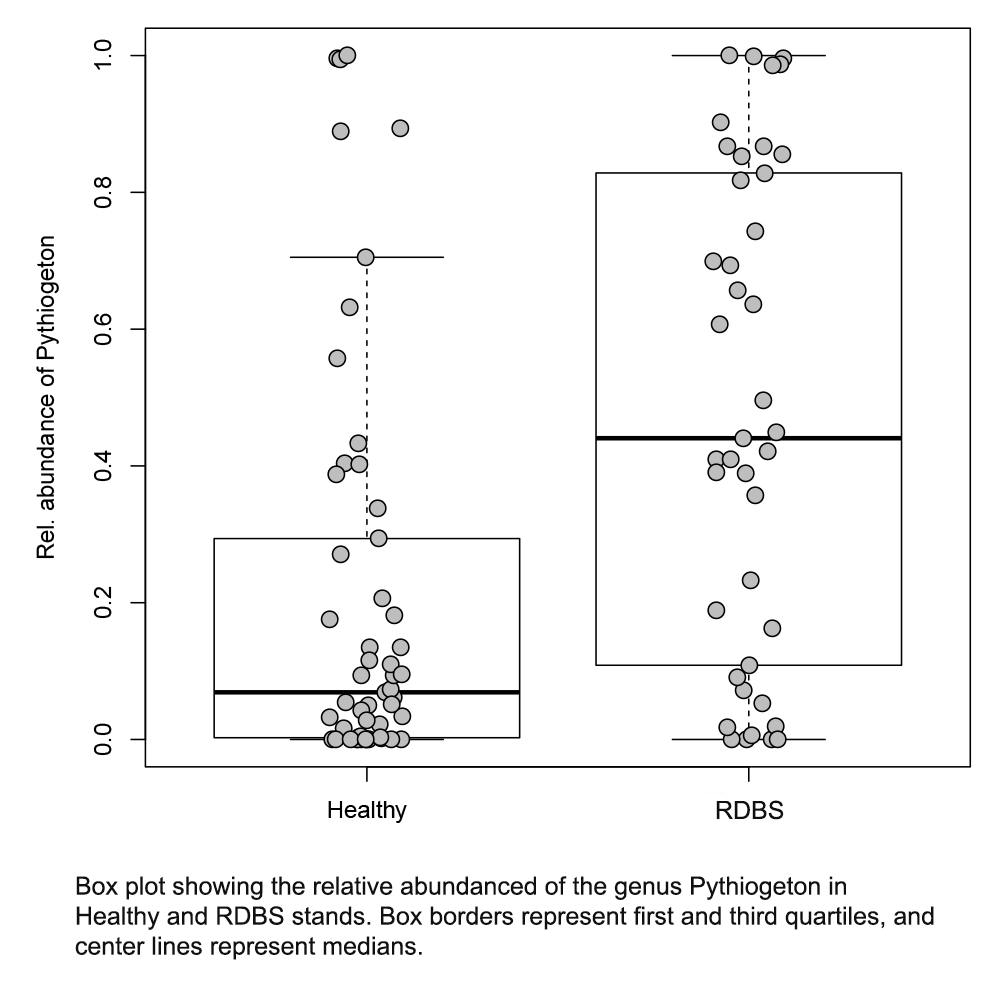

Supplement: Supplementary file 5 [file Image_2.TIF]
